# Epicardial Adipose Tissue (EAT) Thickness Is Associated with Cardiovascular and Liver Damage in Nonalcoholic Fatty Liver Disease

**DOI:** 10.1371/journal.pone.0162473

**Published:** 2016-09-14

**Authors:** Anna Ludovica Fracanzani, Giuseppina Pisano, Dario Consonni, Silvia Tiraboschi, Andrea Baragetti, Cristina Bertelli, Giuseppe Danilo Norata, Paola Dongiovanni, Luca Valenti, Liliana Grigore, Tatiana Tonella, Alberico Catapano, Silvia Fargion

**Affiliations:** 1 Department of Pathophysiology and Transplantation, Ca’ Granda Foundation IRCCS Maggiore Policlinico Hospital, University of Milan, Milan, Italy; 2 Epidemiology Unit, Ca’ Granda Foundation IRCCS Maggiore Policlinico Hospital, University of Milan, Milan, Italy; 3 Department of Pharmacological and Biomolecular Sciences, University of Milan and Centro Studi Aterosclerosi Milan, Milan, Italy; 4 Centro Studi Aterosclerosi, Bassini Hospital, Milan, Italy; 5 Cardiovascular Medicine Unit, Ca’ Granda Foundation IRCCS Maggiore Policlinico Hospital, Milan, Italy; 6 Department of Pharmacological and Biomolecular Sciences, University of Milano, and Multimedica IRCCS, Milan, Italy; Universita degli Studi di Verona, ITALY

## Abstract

**Background and Aims:**

Epicardial adipose tissue (EAT) has been proposed as a cardiometabolic and hepatic fibrosis risk factor in patients with non alcoholic fatty liver disease (NAFLD). Aim of this study was to evaluate the role of EAT in NAFLD by analyzing 1) the association between EAT, the other metabolic parameters and the severity of steatosis 2) the relationship between cardiovascular (cIMT, cplaques, E/A), liver (presence of NASH and significant fibrosis) damage and metabolic risk factors including EAT 3) the relationship between EAT and genetic factors strongly influencing liver steatosis.

**Methods:**

In a cross-sectional study, we considered 512 consecutive patients with NAFLD (confirmed by biopsy in 100). EAT, severity of steatosis, carotid intima-media thickness (cIMT) and plaques were evaluated by ultrasonography and results analysed by multiple linear and logistic regression models. Variables independently associated with EAT (mm) were female gender (p = 0.003), age (p = 0.001), BMI (p = 0.01), diastolic blood pressure (p = 0.009), steatosis grade 2 (p = 0.01) and 3 (p = 0.04), fatty liver index (p = 0.001) and statin use (p = 0.03). Variables independently associated with carotid IMT were age (p = 0.0001), hypertension (p = 0.009), diabetes (p = 0.04), smoking habits (p = 0.04) and fatty liver index (p = 0.02), with carotid plaques age (p = 0.0001), BMI (p = 0.03), EAT (p = 0.02),) and hypertension (p = 0.02), and with E/A age (p = 0.0001), diabetes (p = 0.005), hypertension (p = 0.04) and fatty liver index (p = 0.004). In the 100 patients with available liver histology non alcoholic steatohepatitis (NASH) was independently associated with EAT (p = 0.04) and diabetes (p = 0.054) while significant fibrosis with EAT (p = 0.02), diabetes (p = 0.01) and waist circumference (p = 0.05). No association between EAT and PNPLA3 and TM6SF2 polymorphisms was found.

**Conclusion:**

In patients with NAFLD, EAT is associated with the severity of liver and vascular damage besides with the known metabolic risk factors.

## Introduction

Nonalcoholic fatty liver disease (NAFLD) represents an emerging public health problem worldwide. Indeed, it has reached epidemic proportions and is now the most common liver disease in Western Countries, affecting 20%–34% of the general population[1–3].

NAFLD is considered the hepatic manifestation of the metabolic syndrome (MS), as hepatic fat accumulation is a consequence of systemic insulin resistance and hyperinsulinemia although recently it has been proposed that NAFLD may precede metabolic syndrome [4]. Therefore, NAFLD is strongly associated with and is an independent predictor of cardiovascular diseases, including coronary heart disease and stroke[3, 5], which have been reported to be the first cause of death[6–8].

However the heritability can only partially explain the predisposition to progressive atherosclerotic damage in NAFLD. The I148M variant of patatin-like phospholipase domain-containing-3 (PNPLA3) gene which is involved in lipid remodeling, is actually defined as the major common genetic determinant of NAFLD and it is associated with progression to NASH [9], while its role in atherosclerotic damage has so far been reported only in young patients with NAFLD[10].

Ectopic fat accumulation within and around visceral organs such as liver and heart, and in particular epicardial fat [11, 12], considered the best marker of intra-myocardial fat accumulation for its direct contiguity to myocardium[13, 14], is associated to an increased cardiovascular risk [13, 15]. Indeed, EAT thickness has been reported to predict coronary artery disease, left ventricular dysfunction, and atrial fibrillation [13, 15, 16]

Furthermore, EAT may play a pivotal role in the development and progression of both diastolic and systolic heart failure[17–19]. It is possible that epicardial adipose tissue (EAT) which secretes vasoactive molecules that regulate coronary arterial tone, and modulate inflammation [20–24], interferes with the autonomic nervous system determining both cardiovascular and liver damage [25, 26].

A robust association between EAT, the degree of fatty liver and early atherosclerotic damage has been reported only in obese patients with NAFLD either of adult or pediatric age [27, 28]. In addition, EAT was recently correlated with arterial stiffness and liver fibrosis in NAFLD patients [29, 30]. However studies in large cohorts of patients with NAFLD confirming these observations are not available.

Thus, we conducted this study with the aim to evaluate 1) the association between EAT, the other metabolic parameters and the severity of steatosis 2) the relationship between cardiovascular (cIMT, cplaques, E/A), liver (presence of NASH and significant fibrosis) damage and metabolic risk factors including EAT and 3) the relationship between EAT and genetic factors strongly influencing liver steatosis, including PNPLA3 and TMS6SF2.

## Methods

A cross-sectional study was performed. From May 2013 to June 2014 we enrolled 512 consecutive subjects with diagnosis of NAFLD, done at enrolment by ultrasonography and confirmed by biopsy in 100. Two hundred and eighteen patients were diagnosed at the IRCCS Ca’ Granda, Metabolic Liver Disease outpatient service, and 294 at the Bassini Hospital from a cohort studied for the prevention of dyslipidemia and cardiovascular disease.

Subjects with viral B and C hepatitis, Wilson's disease, a1-antitrypsin deficiency, autoimmune hepatitis, genetic hemochromatosis, and subjects using drugs potentially causing steatosis were excluded from the study.

Subjects were also excluded from this study if a history of cardiovascular disease (CVD) (myocardial infarction, unstable angina, stroke, or cardiovascular revascularization) was recorded.

A complete evaluation of anthropometric, clinical, biochemical parameters was collected at enrollment in the entire cohort of NAFLD including 512 patients. Of these 100 had liver histology. PNPLA3 and TM6SF2 E167K genotypes were studied in 134 patients, 85 belonging to the subgroup of the 100 biopsied subjects and 49 to the non- biopsied patients.

Systolic and diastolic blood pressures were measured twice on the same day, and the mean values were used for analysis. The presence of hypertension was defined as having systolic blood pressure over 140 mmHg or diastolic blood pressure over 90 mmHg more than twice or as taking anti-hypertensive medication. Laboratory examinations included serum aspartate aminotransferase (AST), alanine aminotransferase (ALT), gamma-glutamyl transpeptidase (GGT), triglyceride, high-density lipoprotein cholesterol, low-density lipoprotein cholesterol, total cholesterol, fasting glucose. Blood samples were collected from all participants after a 12-h overnight fast. Diabetes mellitus was diagnosed based on the American Association of Diabetes criteria[31], whereas the MS defined according to the ATP III criteria[32, 33].

Cardiovascular risk was calculated using individual score developed in the Italian population (Progetto Cuore). This was based on continuous values for some risk factors such as age, total serum cholesterol, HDL and systolic blood pressure. Anti-hypertensive therapy is also included in the assessment[34].

Average alcohol daily consumption (in grams of pure ethanol) was calculated by multiplying frequency and amount, using beverage specific standard ethanol contents. In all patients, daily alcohol intake was lower than 20 g in females and 30 g in males (confirmed by at least one family member). In all patients, daily alcohol intake had to be lower than 20 g in females and 30 g in males to be included in the study.

According to smoking habits, individuals were categorized into current, former, or never-smokers.

### Ethic statement

The Institutional Review Board “Comitato Etico Milano Area B, Fondazione IRCCS Ca’ Granda Ospedale Maggiore Policlinico” reviewed and approved the study project (586 bis, 2014). This is the first part of the entire project.

All patients gave informed, written consent to participate in the study according to a protocol, conformed to the ethical guidelines of the 1975 Declaration of Helsinki.

### Assessment of fatty liver by ultrasonography (US) and by Fatty Liver Index (FLI)

FLI was calculated according with literature [35] in all NAFLD patients and correlated with EAT.

Two experienced sonographers, who were unaware of the clinical characteristics of the NAFLD patients, performed hepatic ultrasonography (US) in all subjects at enrollment using a Philips (iU22 Healthcare, Bothell, WA, USA) and Esaote (MyLab70, Italy) device. The degree of hepatic steatosis was defined on the basis of accepted criteria (hepatorenal echo contrast, liver brightness, deep attenuation, and vascular blurring [36, 37]) as mild (grade 1), moderate (grade 2) and severe (grade 3).

### Assessment of Carotid Atherosclerosis

Mean carotid arteries intima-media thickness (cIMT), an index of early atherosclerotic process, was determined by high-resolution B-mode US with a 7.5-MHz transducer, as described previously[38–40]. Values of cIMT represent the mean intima-media thickness on left and right sides. For the common carotid arteries, images of the two sides were obtained 2 cm proximal to the dilatation of the carotid bulb; for each subject, were performed 3 measurements on left and right sides. Three experienced sonographers unaware of the clinical and biochemical characteristics of NAFLD patients performed the evaluation of cIMT and carotid plaques. The inter-rater correlation between the repetition of measurements of intima-media thickness was 0.96 mm (p<0.001), with similar averages of the 2 sets of readings. The imaging of the common and internal carotid arteries of both sides are obtained in multiple longitudinal and transverse planes. All regions of interest was studied to verify the presence of plaques that are defined as a focal thickening >1.2 mm at the level of carotid artery.

### Echocardiographic assessment

A single-experienced cardiologist performed a focused two-dimensional transthoracic echocardiography using a commercially-available device (Esaote My lab 30 gold, Italy). Data acquisition was performed with a 3.5-MHz transducer at a depth of 16 cm in the parasternal and apical views. All recordings were digitally stored for off-line analyses by dedicated software, and were later interpreted by the same cardiologist who performed the test. Conventional echocardiographic parameters were measured according to the American Society of Echocardiography (ASE) guidelines[41]. M-mode echocardiograms of the left ventricle were recorded from the parasternal long-axis view guided by two-dimensional image. The following parameters were determined: left ventricular telediastolic internal diameter (LVIDd), interventricular septum, and posterior wall thickness (PWTd). The relative wall thickness was also calculated by the following formula [(2XPWTd)/LVIDd)], as an index of left ventricular geometry pattern. When optimal orientation of LV M-mode ultrasound beam could not be obtained, correctly orientated linear dimension measurements were performed using two-dimensional imaging. The ejection fraction was calculated at the apical four chamber views, the ejection fraction was calculated over five consecutive beats[42]. LV mass was calculated in grams using the following formula: 0.8×(1.04[(LVID + IVS + PWT)3 − LVID3]) +0.6. LVM was normalized for height to the 2.7 power (LVM/H2.7)[43].

Pulsed-wave Doppler images at the level of the mitral valve tips from apical four- chambers two-dimensional views were obtained to measure flow velocities in the peak early diastolic (E-wave) and peak late diastolic (A-wave) phase, and to calculate their ratio (E/A) as measurement of diastolic filling [44]. Each value was obtained as the average of three measurements.

The most accurate evaluation of the epicardial fat of the right ventricle was obtained by measuring EAT in the parasternal long- and short-axis views, with optimal cursor beam orientation in each view. Maximum EAT, (the mean of at least three measures) was obtained during end systole [45].

### Non-invasive fibrosis evaluation

The FIB-4 was calculated according to literature[46], in particular the regression formula was: age (years) x AST [U/l]/(platelets [10^9^/l] x (ALT [U/l])^1/2^).

The NAFLD Fibrosis Score (NFS) was calculated according to literature [47] in particular the regression formula was = -1.675+0.037 x age (years)+ 0.094 x BMI (kg/m^2^) +1.13 x IGF/diabetes (yes = 1,no = 0)+ 0.99 x AST/ALT ratio-0.013 x platelet (x10^9^/l)- 0.66 x albumin (g/dl).

### Liver Histology

Of the entire cohort of 512 NAFLD in 100 patients US-guided liver biopsy was performed for persistently abnormal liver biochemical tests, increased ferritin or, in patients with normal aminotransferases, in the presence of additional risk factors for NASH such as severe insulin resistance, type 2 diabetes mellitus, and metabolic syndrome. Liver biopsy was performed within 4 months from cardiovascular assessment. The Kleiner classification[48] was used to grade steatosis, lobular inflammation, and hepatocellular ballooning, and to stage fibrosis from 0 to 4. NASH was considered to be present when steatosis, ballooning, and lobular inflammation were all present.

### Genotyping

The rs738409 C>G (I148M PNPLA3) and the rs58542926 C>T (E167K, TM6SF2) single-nucleotide polymorphisms were assessed in duplicate by TaqMan 5’-nuclease assays (Life Technologies, Carlsbad, CA). The success rate and reproducibility were >99%; random samples were confirmed by direct sequencing. The genotype distributions were in Hardy-Weinberg’s equilibrium and were performed in 134 patients.

### Statistical analysis

To compare variables in men and women we used the chi-squared (for categorical variables) and the Mann-Whitney or Kruskal-Wallis test (for quantitative variables). We then performed two sets of multiple linear or logistic regression analyses: a) models adjusted only for age and gender; and b) models fully adjusted for all the variables which were statistically significant in age- and gender-adjusted models, but including only one of the variables with a similar role (diabetes and fasting glucose, diastolic/systolic blood pressure and hypertension, BMI and waist circumference). Statistical analyses were performed with Stata 13 (StataCorp. 2013. Stata: Release 13. Statistical Software. College Station, TX: StataCorp LP.)

### Control group

Six hundred forty seven subjects from a large survey of the general population based volunteer study for the evaluation of cardiovascular risk progression (the Progressione delle Lesioni Intimali Carotidee (PLIC) study), representative of the entire PLIC cohort, in whom steatosis had been ruled out by ultrasonography and b) were studied. Subjects with viral B and C hepatitis, Wilson's disease, a1-antitrypsin deficiency, autoimmune hepatitis, genetic hemochromatosis, and subjects using steatogenic drugs as well as subjects with a history of cardiovascular disease (CVD) (myocardial infarction unstable angina, stroke, or cardiovascular revascularization) were excluded. All Controls was analyzed and studied as the NAFLD patients.

## Results

### Characteristics of patients

Steatosis, as evaluated by US, was of grade 1, 2 and 3 in 230 (45%), 210 (41%) and 72 (14%) of the overall series. Given the marked differences between males and females, we analyzed characteristics of the study cohort according to gender Table 1.

**Table 1:** Characteristics of 512 patients with the overall series of NAFLD according to gender.

Variables	NAFLD	Female	Male	p
	(n = 512)	(n = 189)	(n = 314)	
Age (yrs)	61 ±13	63±10	57±12	0.0001
BMI (Kg/m^2^)	28.9 ±4	29.6±5	28.5±3	0.005
Waist circumference (cm)	100 ±10	97±11	101±9	0.001
Fasting glucose (g/L)	96 ±40	99±61	100±19	0.1
Total Cholesterol (mg/100 mL)	199±40	208±38	196±40	0.004
HDL Cholesterol (mg/100 mL)	54±15	60±15	50±13	0.0001
Triglycerides (mg/100 mL)	130 ±78	123±61	135±86	0.4
AST (U/L)	22± 15	23±13	29±18	0.0001
ALT (U/L)	23 ± 14	25±18	35±23	0.0001
GGT (U/L)	27± 43	32±41	49±45	0.0001
Systolic blood pressure (mmHg)	132 ±16	133±18	132±15	0.8
Diastolic blood pressure (mmHg)	81±9	81±9	80±10	0.6
Hypertension n, (%)	240 (47)	98 (52)	66 (46)	0.2
Diabetes n, (%)	82 (16)	32 (17)	47 (15)	0.4
Metabolic Syndrome n, (%)	215 (42)	85 (45)	125 (40)	0.9
CR score 10-year %	8.4±8	5.8±6	9.4±9	0.0001
Smoke n, (%)	189 (37)	60 (32)	125 (41)	0.05
Ultrasonographic steatosis grade n, (%)				
1	230 (45)	110 (58)	120 (38)	0.001
2	205 (41)	72 (32)	143 (46)	
3	70 (14)	20 (10)	50 (16)	
Fatty Liver Index (FLI)	60±23	55.7±26	62.8±21	0.001
**Cardiovascular parameter**				
cIMT (mm)	0.78±0.19	0.78±0.19	0.78±0.20	0.8
cplaques n, (%)	194 (38)	72 (38)	122 (39)	0.9
EAT (mm)	5.5±3.0	6.2±3.0	5.4±2.9	0.0001
E/A	0.97±0.33	0.91±0.32	1.01±34	0.0001
LVM	184±76	164±68	200±78	0.001
**Non-invasive fibrosis score**				
FIB4 score	1.36±0.9	1.27±0.9	1.4±0.8	0.1
NFS score	-1.55±1.4	-1.54±1.4	-1.56±1.4	0.9
**Liver Histology**°				
NASH n, (%)	67/100 (67)	19/25 (76)	48/75 (64)	0.01
Steatosis grade 3 n, (%)	28/100 (28)	8/25 (32)	20/75 (27)	0.5
Fibrosis stage >2 n, (%)	32/100 (32)	13/25 (52)	19/75 (25)	0.01
Genotype*				
PNPLA3 genotype GG n (%)	26/134 (19)	11/36 (31)	15/106(14)	0.4
TM6SF2 genotype EK+K, n (%)	29/134 (22)	5/34 (15)	24/99 (24)	0.2

mean ±SD; cIMT carotid Intima-media thickness, cplaques = carotid plaques, EAT = epicardial adipose tissue thickness, LVM = left ventricular mass, CR score = Cardiovascular risk score

°Liver histology available in 100 patients

*PNPLA3 and TM6SF2 genotypes available in 134 patients

Male patients were younger, had significantly lower BMI, but higher waist circumference, similar prevalence of hypertension, diabetes and metabolic syndrome than females. The analysis of cardiovascular parameters showed that male patients had a lower EAT, higher diastolic dysfunction (E/A), and LV mass, and, limited to patients with available liver histology, also a lower prevalence of NASH and fibrosis stage >2.

In our series most of the NAFLD women (86%) were over 50 years old. To avoid the possible role of menopausal status in the analyses we evaluated the degree of liver damage in women according to age > or < 50 years. Non-invasive fibrosis score significantly differed in women below or over 50 years (FIB-4 0.78±0.5 vs 1.34±0.7, p = 0.0001, and NFS -3.06±0.97 vs -1.36±1.3,p = 0.0001), while EAT (5.7±2.5 vs 6.2±3, p = 0.2), fatty liver index (51.4±30 vs 55.0±26, p = 0.7), fibrosis >2 (30%vs 61%,p = 0.2) and NASH (85% vs 72%, p = 0.3) did not differ significantly.

The characteristics of patients according to tertiles of EAT are presented in Table 2. A significant higher BMI and waist circumference, prevalence of diabetes, metabolic syndrome, cardiovascular and liver damage were observed across the tertiles.

**Table 2:** Characteristics of 512 patients with NAFLD divided according to EAT tertile.

Variables	NAFLD	EAT	EAT	EAT	p^
	(n = 512)	Tertile1	Tertile 2	Tertile 3	
		(<4.1 mm)	(4.1–6.8 mm)	(≥ 6.9 mm)	
Age (yrs)	61 ±13	57±15	58±13	62±12	0.004
BMI (Kg/m^2^)	28.9 ±4	27.3±3.7	29.1±3.5	30.3±4.4	0.0001
Waist circumference (cm)	100 ±10	96±10	101±9	103±11	0.0001
Fasting glucose (g/L)	96 ±40	96±15	97±21	106±22	0.0001
Total Cholesterol (mg/100 mL)	199±40	202±40	197±42	201±37	0.6
HDL Cholesterol (mg/100 mL)	54±15	55±17	52±14	55±14	0.8
Triglycerides (mg/100 mL)	130 ±78	132±88	132±79	126±65	0.4
AST (U/L)	22± 15	26±16	27±16	26±18	0.9
ALT (U/L)	23 ± 14	32±23	32±24	34±17	0.1
GGT (U/L)	27± 43	40±38	44±43	42±49	0.6
Systolic blood pressure (mmHg)	132 ±16	128±13	133±16	135±18	0.0001
Diastolic blood pressure (mmHg)	81±9	78±10	81±8	83±10	0.0001
Hypertension n, (%)	240 (47)	72 (42)	79 (46)	89 (52)	0.04
Diabetes n, (%)	82 (16)	13(8)	28 (16)	41(24)	0.0001
Metabolic Syndrome n, (%)	215 (42)	52 (31)	68 (40)	95(56)	0.0001
CR score 10-year %	8.4±8	7.2±8.1	8.3±8.3	8.7±7.6	0.1
Smoke n, (%)	189 (37)	57(34)	64(38)	71(42)	0.1
Ultrasonographic steatosis grade n, (%)					
1	230 (45)	92 (55)	74 (44)	64 (38)	Ref
2	205 (41)	61 (36)	69 (41)	75 (44)	0.015
3	70 (14)	15 (9)	25 (15)	30 (18)	0.003
Fatty Liver Index (FLI)	60±23	52±25	61±22	66±22	0.0001
**Cardiovascular parameter**					
cIMT (mm)	0.78±0.19	0.75±0.20	0.80±0.20	0.79±18	0.04
cplaques n, (%)	194 (38)	52 (31)	66 (39)	76 (45)	0.004
E/A	0.97±0.33	1.04±0.38	0.99±0.34	0.88±0.26	0.0001
LVM	184±76	180±73	187±69	185±85	0.6
**Non-invasive fibrosis score**					
FIB4 score	1.36±0.9	1.32±1.0	1.32±0.8	1.43±0.9	0.3
NFS score	-1.55±1.4	-1.83±1.3	-1.59±1.4	-1.22±1.4	0.0001
**Liver Histology**°					
NASH n, (%)	67/100 (67)	14/28 (52)	31/41 (76)	22/31 (88)	0.005
Steatosis grade n, (%)					
1	25/100 (25)	10/28 (36)	10/41 (24)	5/31(16)	Ref
2	47/100 (47)	12/28 (43)	20/41 (49)	15/31 (48)	0.17
3	28/100 (28)	6/28 (21)	11/41 (27)	11/31 (35)	0.08
Fibrosis stage >2 n, (%)	32/100 (32)	2/28 (7)	13/41 (32)	17/31(55)	0.0001
Genotype*					
PNPLA3 genotype GG n (%)*	26/134 (19)	9/43 (20)	8/56 (14)	9/35 (26)	0.1
TM6SF2 genotype EK+K, n (%)*	29/134 (22)	10/45(22)	14/56(25)	5/33(15)	0.5

° Liver histology available in 100 cases

* PNPLA3 and TM6SF2 genotypes available in 134 cases

^ p = for trend

When we analyzed the characteristics of NAFLD in the subgroup of the 100 biopsy-proven and in the subgroup with PNPLA3 and TM6SF2 genotyping, as expected, patients who underwent liver biopsy had worse metabolic and liver profile while the parameters of the genotyped patients were similar to that of the entire cohort.

Patients with NAFLD had a significant higher levels of EAT that control subjects of general population in addition to all others metabolic, anthropometric, and cardiovascular damage parameters (S1 Table).

### Association of EAT with severity of liver steatosis, by US and Fatty Liver Index, (FLI) and with metabolic parameters

**Fig 1:**
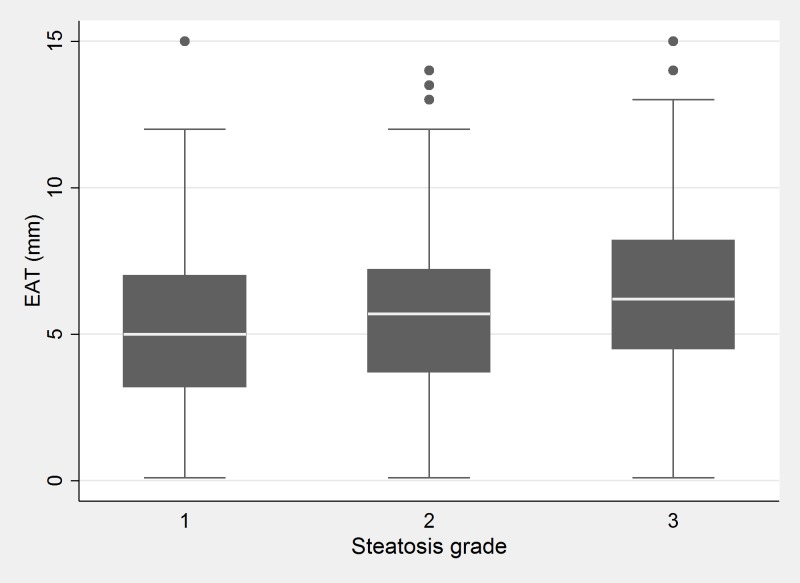
Association between EAT and US degree of steatosis. EAT according to US fatty liver grade, (p<0.001 for trend).

The association between EAT and US degree of steatosis at univariate analysis is shown in Fig 1 (p<0.001 for trend).

Results of the association between epicardial adipose tissue (EAT, mm) metabolic and clinical variable, ultrasonographic steatosis grades and FLI are reported in Table 3. Age, male gender, BMI, diastolic blood pressure, the degree of ultrasonographic steatosis, FLI and the use of statin were independently associated with EAT. Waist circumference used instead of BMI in multivariable model was found independently associated with EAT (coefficient 0.06, 95% C.I. 0.03–0.09, p = 0.0001).

**Table 3:** Association between epicardial adipose tissue (EAT, mm) metabolic and clinical variables and ultrasonographic steatosis grades. Results of multiple linear regression analyses, age- and gender-adjusted and fully adjusted*.

	Age and gender adjusted model			Fully adjusted model *		
Variables	Coefficient	95% C.I.	P	Coefficient	95%C.I.	P
Age (yrs)	-	-	**-**	0.21	0.11, 0.42	**0.04**
Female gender	-	-	**-**	0.47	0.20,0.74	**0.001**
BMI (Kg/m^2^)	0.2	0.1, 0.3	**0.0001**	0.18	0.12, 0.24	**0.0001**
Waist circumference (cm)°	0.08	0.06, 0.11	**0.0001**	0.06	0.03, 0.09	**0.0001**
Fasting glucose (g/L)	0.23	0.10, 0.37	**0.001**	Not included	-	-
Systolic blood pressure (mmHg)	0.29	0.12, 0.45	**0.01**	-0.30	-0.33,0.17	0.7
Diastolic blood pressure (mmHg)	0.63	0.36, 0.91	**0.0001**	0.46	0.11,0.82	**0.009**
Hypertension	0.13	-0.41, 0.68	0.6			
Diabetes	1.1	0.37, 1.79	**0.003**	0.54	-0.17,1.2	0.14
Metabolic Syndrome	1.1	0.53, 1.63	**0.0003**	Not included	**-**	**-**
Smoke	0.76	0.22, 1.31	**0.006**	0.45	-0.07,0.50	0.09
Ultrasonographic Steatosis grade						
Reference: 1	Ref	Ref		-	-	-
2	1.1	0.47, 1.58	**0.0001**	0.6	-0.02,1.3	0.058
3	1.8	1.04, 2.59	**0.001**	1.42	0.4, 2.4	**0.007**
Fatty liver index (FLI)	0.04	0.02, 0.05	**0.0001**	0.02	0.01, 0.04	**0.0001**
Statin use	0.6	-0.03, 1.3	0.06	0.8	0.06,1.5	**0.03**

* Fully adjusted = each variable adjusted for the others except when indicated.

° In the fully adjusted model we included either waist circumference or BMI

The multivariate analysis of the association of EAT level higher 4 mm (the median values of control subjects without steatosis) in the overall series including NAFLD patients and Controls indicated that EAT was significantly associated with age, female gender, BMI, waist circumference, smoke habits, steatosis, cplaques and E/A (S2 Table).

### Relationship between cardiovascular damage (cIMT, cplaques, E/A) and metabolic risk factors including EAT

**Fig 2:**
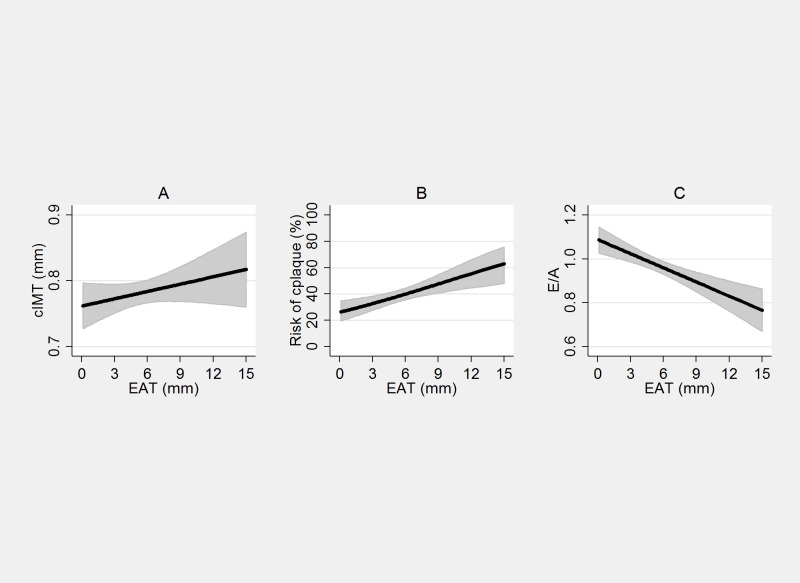
Association between EAT and cardiovascular parameters. EAT (unit) and cIMT (panel A), coefficient β: 0.005, (95% C.I. 0.001, 0.009), p = 0.007, EAT and cplaques (panel B) O.R. 1.10 (95%C.I. 1.04–1.18), p<0.0001, EAT and E/A (panel C) coefficient β -0.02, (95%C.I. -0.03, -0.01), p = <0.0001).

The associations between EAT and cardiovascular damage parameters (cIMT, cplaques, E/A) at univariate analysis are shown in Fig 2. A significant linear increase of cIMT and risk of plaques and a decrease of E/A with increasing EAT was present.

The multiple linear (cIMT, E/A) and logistic regression (cplaques) analyses, age- and gender-and fully adjusted models are shown in Table 4. cIMT in the fully adjusted model was independently associated with age, hypertension, diabetes, FLI and smoking habits, cplaques with age, BMI, EAT and hypertension, and E/A with age, diabetes, FLI and hypertension.

**Table 4:** Association of parameters of cardiovascular damage (cIMT (mm), cplaques and E/A) with EAT and metabolic, clinical variables and ultrasonographic steatosis grades. Results of multiple linear (cIMT, E/A) and logistic regression (cplaques) analyses, age- and gender-adjusted and fully adjusted models*.

	Age and gender adjusted model			Fully adjusted model *		
Variables	Coefficient	95% C.I.	P	Coefficient	95%C.I.	P
	**cIMT**			**cIMT**		
Age (yrs)	-	-	**-**	0.04	0.03, 0.06	**0.0001**
Male gender	-	-	**-**	-0.19	-0.04, 0.05	0.18
BMI (Kg/m^2^)	0.005	0.0001, 0.008	**0.03**	0.004	-0.007, 0.008	0.9
Waist circumference (cm)	0.002	0.0007, 0.004	**0.005**	0.002	0.0002, 0,004	**0.03**
EAT (mm)	0.0008	-0.004, 0.006	0.7	-0.004	-0.011, 0.002	0.2
Fasting glucose (g/L)	0.01	0.002,0.02	**0.02**	Not included	**-**	-
Hypertension	0.06	0.03, 0.09	**0.001**	0.05	0.01, 0.09	**0.009**
Diabetes	0.07	0.03, 0.11	**0.002**	0.05	0.003,0.1	**0.04**
Metabolic Syndrome	0.02	-0.09, 0.065	0.1	**-**	**-**	**-**
Smoke	0.04	0.007,0.78	**0.02**	0.05	0.002, 0.09	**0.04**
Ultrasonographic Steatosis grade						
1	Ref	Ref		Ref	Ref	
2–3	1.14	0.02,1.25	**0.05**	-0.01	0.2, -0.04	0.2
Fatty liver index (FLI)	0.001	0.004, 0.007	**0.0008**	0.001	0.0001, 0.002	**0.02**
Statin use	0.03	-0.01, 0.07	0.2	0.04	-0.009, 0.08	0.1
	**cplaques**			**cplaques**		
	O.R.	95%C.I.	**P**	O.R.	95%C.I.	**P**
Age	-	-	**-**	2.3	1.73, 2.80	**0.0001**
Male gender	-	-	**-**	0.0	0.40,1.06	0.09
BMI (Kg/m^2^)	0.98	0.93,1.04	**0.6**	0.93	0.88,1.0	**0.03**
Waist circumference (cm)°	1.01	0.99, 1.03	**0.2**	0.97	0.94, 0.99	**0.04**
EAT (mm)	1.08	1.01,1.17	**0.02**	1.10	1.0–1.12	**0.02**
Smoke	1.4	0.90, 2.1	0.1	-	-	-
Diabetes	1.5	0.88, 2.6	0.1	0.99	0.54,1.8	0.98
Hypertension	1.8	1.18, 2.7	**0.006**	2.1	1.2, 3.5	**0.005**
Metabolic Syndrome	0.88	0.56,1.34	0.6	-	-	**-**
Ultrasonographic Steatosis grade						
1	Ref	Ref		-	-	**-**
2–3	3.6	0.89,15	0.07	-	-	**-**
Fatty liver index	0.99	0.98, 1.1	0.5	-	-	**-**
Statin use	0.5	0.1,1.0	**0.03**	1.1	0.6, 2.03	0.7
	**E/A**			**E/A**		
	O.R.	95%C.I.	**P**	O.R.	95% C.I.	**P**
Age (yrs)	-	-	**-**	-0.09	-0.11, -0.06	**0.0001**
Male gender	-	-	**-**	0.03	-0.04, 0.11	0.4
BMI (Kg/m^2^)	-0.007	-0.013, 0.004	**0.04**	0.003	-0.009, 0.2	0.6
Waist circumference (cm)°	-0.003	-0.007,-0.001	**0.008**	0.003	-0.02, 0.008	0.2
EAT (mm)	-0.12	-0.021, -0.003	**0.007**	-0.008	-0.02, 0.11	0.1
Diabetes	-0.11	-0.18,-0.03	**0.005**	-0.01	-0.02, 0.03	**0.005**
Hypertension	-0.08	-0.12, -0.017	**0.01**	-0.05	-0.11, 0.1	**0.04**
Metabolic Syndrome	-0.07	-0.13,-0.16	**0.01**	Not included	-	**-**
Smoke	-0.002	-0.05, 0.05	0.9	0.006	-0.05, 0.07	0.8
Ultrasonographic Steatosis grade						
1	Ref	Ref	-	-	-	-
2–3	-0.02	-0.17, 0.11	0.9	-	-	-
Fatty liver index (FLI)	-0.002	-0.003, -0.001	**0.0003**	-0.003	-0.005, -0.001	**0.004**
Statin use	-0.01	-0.08, 0.06	0.7	0.06	-0.7, 0.08	0.8

* Fully adjusted = each variable adjusted for the others except when indicated.

cIMT carotid Intima-media thickness, cplaques = carotid plaques, EAT = epicardial adipose tissue thickness

° In the fully adjusted model we included either waist circumference or BMI

Patients with cplaques had significant higher values of EAT than those without (6.1±3 v.s.5.0±2.9, p = 0.0001).

In addition we evaluated the association between EAT and cplaques separately in men and women, the O.R. in man was 1.19 (95% C.I. 1.05–1.35, p = 0.005) and in woman 1.04 (95% C.I. 091–1.19, p = 0.55), however p for gender-EAT interaction was not significant (p = 0.14).

### Relationship between non-invasive fibrosis score and EAT

To evaluate the relationship between EAT and NFS we performed a linear regression analysis and we found a significant positive correlation (slope +0.4 mm C.I.0.19–0.60, p<0.001) for each unit increase of NFS. We made the similar analysis with FIB4 (log-e transformation because of the highly skewed distribution) and we also found a significant correlation with EAT (slope +0.58, C.I. 0.03–1.14, p = 0.039).

### Relationship between histological liver damage (presence of NASH and fibrosis >2) and metabolic risk factors including EAT

The relationship was evaluated in the 100 patients who underwent liver biopsy.

A logistic regression analysis, age- and gender-and fully adjusted model is shown in Table 5. steatosis of grade 3 was independently associated only with age; NASH was independently associated with EAT and diabetes and significant fibrosis (>2) with waist circumference, EAT and diabetes.

**Table 5:** Association of parameters of liver damage (steatosis grade 3, NASH and fibrosis>2) and EAT and metabolic and clinical variables. Results of logistic regression analyses age- and gender-adjusted and fully adjusted models (analysis performed in 100 patients with liver histology).

	Age and gender adjusted model			Fully adjusted model *		
Variables	Coefficient	95% C.I.	p	Coefficient	95%C.I.	P
	**Steatosis grade 3**			**Steatosis grade3**		
Age (yrs)	**-**	**-**	**-**	0.8	0.7,0.9	**0.02**
Male gender	**-**	**-**	**-**	-	-	**-**
BMI (Kg/m^2^)	1.2	0.99,1.4	0.06	-	-	-
Waist circumference (cm)°	1.04	0.95,1.1	0.4	-	-	-
EAT (mm)	1.1	0.89,1.2	0.5	-	-	-
ALT (UI)	0.04	0.01,0.08	0.02	1.4	1.003,1.88	**0.05**
Diabetes	4.4	0.9, 24	0.08	-	-	-
Metabolic Syndrome	1.4	0.5,3.9	0.5	-	-	-
Fatty liver Index	1.0	0.98, 1.03	0.7	-	-	-
Statin use	1.04	0.2,4.5	0.9	-	-	-
	**NASH**			**NASH**		
Age (yrs)	-	-	-	0.24	0.03, 0.06	0.4
Male gender	-	-	**-**	4.90	0.68,18	0.13
BMI (Kg/m^2^)	0.9	1.0, 1.1	0.08	1.07	0.92,1.3	0.3
Waist circumference (cm)°	1.1	0.9, 1.0	0.1	-	-	-
EAT (mm)	1.3	1.1, 1.6	**0.01**	1.70	1.1,2.0	**0.01**
Diabetes	3.6	1.0, 17	**0.05**	35	0.99, 1.0	**0.054**
Fatty liver index	1.01	0.98, 1.04	0.3	1.02	0.96, 1.08	0.4
Statin use	1.36	0.3, 6.1	0.6	-	-	
	**Fibrosis >2**			**Fibrosis >2**		
Age (yrs)	-	-	-	1.04	0.4, 8.9	0.5
Male gender	-	-	**-**	1.70	0.50, 2.09	0.9
BMI (Kg/m^2^)	1.1	1.0, 1.1	**0.03**	0.90	0.7,1.2	0.6
Waist circumference (cm)°	1.1	1.1, 1.2	**0.004**	1.10	1,1.2	**0.05**
EAT (mm)	1.4	1.1, 1.8	**0.04**	1.46	1.06, 2.0	**0.02**
Diabetes	1.8	0.6, 5.2	0.2	9.5	1.4, 67	**0.02**
Steatosis						
Fatty liver index	1.1	1.0,1.6	**0.04**	1.02	0.96,1.08	0.4
Statin use	1.7	0.5,6.1	0.4	-	-	-

* Fully adjusted = each variable adjusted for the others except when indicated.

° In the fully adjusted model we included either waist circumference or BMI

When patients were stratified according to the presence of fibrosis (stage F0-F2, n = 61 (61%), F3-F4, n = 39 (39%)), EAT values were higher in patients with more severe fibrosis (6.8, 5.5–7.4 vs. 4.9, 3.1–6.7 mm, in F3-F4 and F0-F2 respectively; p = 0.02).

The mean value of EAT in patients with or without NASH (5.9± 2.5 vs 4.0±, 2.4,p = 0.001) and with or without fibrosis >2 (6.9±2.3 vs 4.7±2.5, p = 0.0001) was significantly higher.

### Association between EAT, PNPLA3 and TM6SF2 polymorphisms

In Tables 1 and 2 are reported results of the prevalence of PNPLA3 and TM6SF2 polymorphisms, evaluated in 134 patients with NAFLD. We did not observe significant differences of EAT value in patients homozygous or heterozygous for the two polymorphisms. Patients with CG+GG genotypes of PNPLA3 and patients with EK+K genotypes of TM6SF2 had higher prevalence of NASH (70 and 76% vs 30 and 24%) and of Fibrosis ≥2 (53 and 71% vs 47 and 29) in comparison of patients wild type, but data did not reach statistical significance possibly for the low number of cases.

## Discussion

In the present study we analyzed in a large series of subjects with NAFLD whether EAT correlates with cardiovascular and liver damage and we demonstrated that EAT is associated both with subclinical atherosclerosis and NASH and significant fibrosis independently of other metabolic confounders (BMI, waist circumference, diabetes, hypertension....) and that there is a significant relationship between increased value of EAT and cardiovascular and liver damage and metabolic alterations. The relationships between EAT, and cardiovascular damage has been recently reported in selected patients series[16, 27–30, 49–52], however this is the first study, to our knowledge, performed in a large series of well characterized NAFLD patients in whom we demonstrated a significant correlation between EAT values and atherosclerosis as documented by the independent association with cplaques. Previous observations suggested an association between epicardial adiposity and the presence and severity of coronary artery disease[17–19, 24, 53], and more recently with vascular stiffness, another subclinical sign of carotid atherosclerosis [29, 54]. Thus EAT contributing to the development and progression of atherosclerosis, may represent a measurable risk factor and in future a modifiable therapeutic target as recently suggested [55].

Iacobellis et al. [28] showed that obese patients with severe fatty liver infiltration have a high amount of epicardial fat accumulation, we confirm these results in a large series of non-obese, overweight NAFLD subjects. In addition we observed a parallel trend between EAT and liver fat suggesting that ectopic fat may be simultaneously present in different organs inducing organ specific damage, as recently hypothesized[56, 57]. In line with this is the observation that subclinical vascular damage, defined as an increase of cIMT, paralleled the severity of steatosis evaluated at ultrasonography and the fatty liver index, reported to be a good indicator of hepatic steatosis [35].

We also showed an independent association between EAT, NASH and significant liver fibrosis, confirming recent results [30, 49]. Interestingly, in these patients we observed a significant independent association between EAT and atherosclerosis and between EAT and liver damage, supporting the hypothesis that the cytokines/adipocytokines secreted by epicardial adipose tissue, acting as a paracrine tissue, favor the local inflammation in vessel and liver, being responsible for the damage of these districts. The inflammatory state that characterizes NAFLD/NASH might be able to act systemically, affecting the homeostasis of different organs as demonstrated for systemic atherosclerosis [58] and kidney [59]. Moreover in our series of NAFLD patients when we performed the analysis adjusted for age and gender, which was necessary because of the strong effect of age and the differences between genders, we observed a significant association between EAT and metabolic syndrome, as extensively reported in literature[60]. These results were confirmed in fully adjusted multivariate analysis in which the single components of metabolic syndrome were not included (data not shown).

In our study, given the large series of subjects analyzed, we could arbitrarily define a value of EAT (≥ 6 mm) able to define NAFLD patients at higher risk of more severe vascular and liver damage. This value could represent an implement to identify subjects requiring a more intensive hepatic and vascular diagnostic approach and follow-up, and represent an additional tool for the stratification of cardiovascular risk in general population as recently proposed by our group[61]. This is reinforced by the results obtained by comparing the value of EAT of NAFLD patients with those of a control group of general population in which the level of EAT was significantly lower.

Finally we analyzed whether polymorphisms of PNPLA3, the most important known genetic determinant of NAFLD and highly associated with severity of hepatic damage and fibrosis, influence the entity of EAT, but we did not find any significant association between this genetic marker and EAT, carotid atherosclerosis (cIMT or cplaques) despite patients who carried GG genotype had more severe liver disease. However, while the role of PNPLA3 in the severity of liver injury has been well documented, its role on vascular damage is still debated[62, 63], and needs to be validated in a larger cohort of subjects. We obtained similar results when we evaluated the TM6SF2 polymorphisms. In fact we did not observe any difference in the prevalence of TM6SF2 polymorphisms at increasing values of EAT. However we cannot rule out that this may be due to the relatively small number of patients studied.

Strengths of this study include the large series of well characterized NAFLD subjects who had abdominal ultrasonographic, carotid and heart assessment performed at the same time, thereby allowing to compare fat accumulation in different sites, and to analyze their association with the cardio-metabolic status and severity of liver damage.

Limitations were related to the lack of direct assessment of visceral fat accumulation by NMR, which was precluded by the large series of patients considered. Furthermore, liver biopsy was available in a minority of NAFLD patients, due to the study design and ethical reasons, however if the reference values of EAT (≥6 mm) will be confirmed in larger series of biopsy proven NAFLD, EAT could represent a non invasive marker of inflammation and used to better characterize the presence of NASH. Finally, we could not quantify inflammatory molecules (e.g., tumor necrosis factor-alpha, cytokines, adipokines…) released by epicardial fat which potentially could have helped to explain the relationship among fatty liver, EAT, and cardiovascular damage.

In conclusion, our results suggest that epicardial fat deposition is part of the same metabolic disorders associated with the development of fatty liver in individuals with insulin resistance. High EAT may represent a marker of the severity of this condition, as highlighted by its association with NASH, liver fibrosis and cplaques, and may also be involved in the progression of target organ damage, especially at the level of heart, vessels and liver.

We further emphasize the need for non-invasive cardiovascular assessment and for a more accurate study of liver damage in patients with steatosis given the high risk of developing fibrosis. Longitudinal studies are required to establish whether EAT represents an independent predictor of cardiovascular events, the main cause of death of patients with NAFLD, and whether may help to identify patients at risk of progressive disease.

## Supporting Information

S1 Table Characteristics of the 647 controls and 512 patients with NAFLD. Univariate analysis (Mann-Whitney test) and linear multiple regression analysis adjusted for age and gender.
(DOCX)

S2 Table Variables significantly associated with EAT higher than 4.0 mm (median of controls without fatty liver). Logistic regression analysis and at multivariate analysis in the overall series (NAFLD and Controls).
(DOCX)
