# Supplementary material for: Epicardial Adipose Tissue (EAT) Thickness Is Associated with Cardiovascular and Liver Damage in Nonalcoholic Fatty Liver Disease
Source: PLoS One. 2016 Sep 14;11(9):e0162473. doi: 10.1371/journal.pone.0162473 (PMC5023162; doi:10.1371/journal.pone.0162473)
Supplement: S1 Table — Univariate analysis (Mann-Whitney test) and linear multiple regression analysis adjusted for age and gender. (DOCX) [file pone.0162473.s001.docx]

S1 Table. Characteristics of the 647 controls and 512 patients with NAFLD at univariate analysis (Mann-Whitney test) and linear multiple regression analysis adjusted for age and gender.

| **Variables** | **No NAFLD**  **n= 647** | **NAFLD**  **n= 512** | **p** | **Adjusted for age and gender**  **p** |
| --- | --- | --- | --- | --- |
| Age (yrs) | 66 (56-73) | 61 (50-70) | .0001 | - |
| Gender (M) | 249 (38) | 319 (62) | .0001 | - |
| BMI (Kg/m^2^) | 25 (23-28) | 28 (26-31) | .0001 | .0001 |
| Waist circumference (cm) | 90 (81-95) | 100 (94-106) | .0001 | .0001 |
| Fasting glucose (g/L) | 90 (83-97) | 96 (88-107) | .0001 | .0001 |
| Total Cholesterol (mg/100 mL) | 202 (181-227) | 199 (174-225) | .11 | .55 |
| HDL Cholesterol (mg/100 mL) | 63 (53-74) | 49 (40-61) | .0001 | .0001 |
| Triglycerides (mg/100 mL) | 81 (64-110) | 122 (86-164) | .0001 | .0001 |
| AST (U/L) | 19 (16-21) | 22 (18-28) | .0001 | .0001 |
| ALT (U/L) | 16 (13-20) | 23 (17-36) | .0001 | .0001 |
| GGT (U/L) | 17 (13-24) | 27 (18-45) | .001 | .0001 |
| Systolic blood pressure (mmHg) | 125 (120-140) | 130 (120-140) | .0001 | .0001 |
| Diastolic blood pressure (mmHg) | 80 (70-80) | 80 (75-90) | .0001 | .0001 |
| Hypertension | 213 (33) | 243 (47) | .0002 | .0001 |
| Diabetes | 26 (4) | 80 (16) | .001 | .0001 |
| Metabolic Syndrome | 103 (16) | 174 (34) | .0001 | .0001 |
| CR score 10-year % | 4.8 (1.9-9.5) | 5.5 (2.4-11.0) | .02 | .001 |
| Smoke habits | 145(23) | 189 (37) | .001 | .001 |
| **Cardiovascular assessment** |  |  |  |  |
| cIMT (cm) | 0.74 (0.62-0.86) | 0.77 (0.65-1.75) | .02 | .001 |
| cplaques | 251 (39) | 194 (38) | .61 | .88 |
| EAT (mm) | 4.0 (2.0-5.9) | 5.5 (3.5-7.2) | .0001 | .0001 |
| E/A | 0.94 (0.74-1.27) | 0.89 (0.72-1.16) | .009 | .001 |
| LVM (g) | 153 (114-210) | 187 (137-246) | .0001 | .002 |

Median (25^th^-75^th^ percentile), n (%)

US=ultrasonography, EAT = epicardial adipose tissue thickness, CR=cardiovascular risk (score), LVM =left ventricular mass
