# Supplementary material for: Epicardial Adipose Tissue (EAT) Thickness Is Associated with Cardiovascular and Liver Damage in Nonalcoholic Fatty Liver Disease
Source: PLoS One. 2016 Sep 14;11(9):e0162473. doi: 10.1371/journal.pone.0162473 (PMC5023162; doi:10.1371/journal.pone.0162473)
Supplement: S2 Table — Logistic regression analysis and at multivariate analysis in the overall series (NAFLD and Controls). (DOCX) [file pone.0162473.s002.docx]

S2 Table. Variables significantly associated with EAT higher than 4.0 mm (median of controls without fatty liver) at logistic regression analysis and at multivariate analysis in the overall series (NAFLD and Controls)

|  | Logistic regression | | | Multivariate | | |
| --- | --- | --- | --- | --- | --- | --- |
| Variables | OR | 95% C.I. | p | O.R | 95% C.I. | p |
| Age (years) | 1.01 | 1.002-1.02 | 0.009 | 1.01 | 1.00-1.02 | **0.001** |
| Female gender | 0.86 | 0.68-1.08 | 0.2 | 0.49 | 0.32-0.74 | **0.02** |
| BMI (Kg/m^2^) | 1.17 | 1.13-1.19 | 0.001 | 1.10 | 1.02-1.18 | **0.008** |
| Waist circumference (cm) | 1.05 | 1.03-1.06 | 0.001 | 1.02 | 1.01-1.06 | **0.05** |
| Smoke habits | 1.53 | 1.16-2.01 | 0.002 | 1.37 | 1.00-1.88 | **0.05** |
| Fasting glucose (g/L) | 1.17 | 1.09-1.27 | 0.0001 | 1.03 | 0.94-1.13 | 0.5 |
| HDL | 0.92 | 0.85-0.98 | 0.02 | 1.02 | 0.90-1.16 | 0.7 |
| Steatosis | 2.76 | 2.13-3.58 | 0.0001 | 1.78 | 1.27-2.53 | **0.001** |
| Diabetes | 2.14 | 1.4-3.27 | 0.0001 | 1.04 | 0.95-1.15 | 0.4 |
| Metabolic syndrome | 2.07 | 1.56-2.74 | 0.0001 | 0.93 | 0.61-1.01 | 0.7 |
| Hypertension | 1.50 | 1.17-1.92 | 0.001 | 1.12 | 0.82-1.54 | 0.45 |
| CR score 10-year % | 1.03 | 1.02-1.04 | 0.001 | 1.02 | 0.98-1.05 | 0.2 |
| **Cardiovascular parameters** |  |  |  |  |  |  |
| cIMT (mm) | 3.2 | 1.7-6.2 | 0.001 | .001 | -.004,0.004 | 0.6 |
| cplaques | 1.54 | 1.21-1.97 | 0.0004 | 1.10 | 1.0-1.12 | **0.05** |
| E/A | 0.43 | 0.31-0.59 | 0.0001 | -.673 | -1.3,-0.12 | **0.04** |

CR=Cardiovascular risk (score), E/A= LVM =left ventricular mass.

Not significant: Total cholesterol, Triglycerides, AST, ALT, GGT

For fasting glucose, systolic and diastolic blood pressure values are reported per 10 units increase.
